# Post-Translational Modifications of Huntingtin: Mechanistic Insights and Therapeutic Opportunities in Huntington’s Disease

**DOI:** 10.3390/ijms262210907

**Published:** 2025-11-11

**Authors:** Xiaoxia Zhang, Shengping Zhang, Chuangui Wang

**Affiliations:** Biomedical Research Institute, School of Life Sciences and Medicine, Shandong University of Technology, Zibo 255049, China; 23410011017@stumail.sdut.edu.cn (X.Z.); spzhang@sdut.edu.cn (S.Z.)

**Keywords:** Huntington’s disease, post-translational modification, Huntingtin

## Abstract

Huntingtin (HTT) is a large, ubiquitously expressed scaffold protein that participates in multiple cellular processes, including vesicular transport, transcriptional regulation, and energy metabolism. The mutant form of HTT (mHTT), characterized by an abnormal polyglutamine (polyQ) expansion in its N-terminal region, is the causative agent of Huntington’s disease (HD), a progressive neurodegenerative disorder. Current therapeutic efforts for HD have primarily focused on lowering HTT levels through gene silencing or promoting mHTT degradation. However, accumulating evidence suggests that post-translational modifications (PTMs) of HTT—such as phosphorylation, ubiquitination, acetylation, and SUMOylation—play pivotal roles in modulating HTT’s conformation, aggregation propensity, subcellular localization, and degradation pathways. These modifications regulate the balance between HTT’s physiological functions and pathological toxicity. Importantly, dysregulation of PTMs has been linked to mHTT accumulation and selective neuronal vulnerability, highlighting their relevance as potential therapeutic targets. A deeper understanding of how individual PTMs and their crosstalk regulate HTT homeostasis may not only provide mechanistic insights into HD pathogenesis but also uncover novel, more specific strategies for intervention. In this review, we summarize recent understanding on HTT PTMs, discuss their implications for disease modification, and outline critical knowledge gaps that remain to be addressed.

## 1. Introduction

Huntington’s disease (HD) is an autosomal dominant neurodegenerative disorder characterized by progressive degeneration of medium spiny neurons (MSNs) in the striatum, leading to motor dysfunction, cognitive impairment, and psychiatric symptoms [1]. A hallmark pathological feature of HD is the accumulation of Huntingtin protein (HTT) aggregates in neuronal nuclei, cytoplasm, and synaptic compartments [2]. These aggregates range from soluble oligomers and protofibrils to large insoluble inclusion bodies and have been implicated in neuronal toxicity [3]. Accumulating evidence suggests that intermediate species formed during the aggregation process, particularly soluble oligomers, are more toxic than mature inclusions [4]. These species interfere with critical cellular functions, including transcription [5], mitochondrial integrity [6], synaptic signaling [7], and proteostasis [8], and neuronal cell death [9]. This is particularly evident in the striatum, where aggregation correlates strongly with selective neuronal vulnerability.

It has been well established that abnormal expansion of CAG trinucleotide repeats in the HTT gene is the major cause of mutant form HTT (mHTT) protein with an extended polyglutamine (polyQ) [10]. The mHTT undergoes profound conformational changes that drive its misfolding and aggregation [11,12]. Although the major cause of HTT aggregation is polyQ expansion, post-translational modifications (PTMs) have emerged as crucial modulators of HTT structure, function, and aggregation propensity. Modifications such as acetylation [13], phosphorylation [14], ubiquitination [15], SUMOylation [16], and palmitoylation [17] have been identified on HTT and shown to regulate its solubility, subcellular localization, and clearance via proteolytic pathways.

Despite these advances, our understanding of the functional landscape of HTT PTMs remains incomplete. Key questions remain regarding the spatiotemporal dynamics of these modifications, their cooperative or antagonistic interactions, and their cell-type-specific effects in the context of HD [18]. Moreover, translating insights from PTM biology into effective therapeutic strategies has proven challenging due to the complexity of HTT regulation and the multifactorial nature of HD pathogenesis.

In this review, we provide a comprehensive overview of the major PTMs identified on HTT/mHTT, their known regulatory enzymes, and their mechanistic roles in modulating mHTT aggregation and pathogenicity. We examine the interplay between HTT/mHTT post-translational modifications and aggregate formation, their role in cytotoxicity, and discuss the therapeutic prospects of targeting PTMs as a strategy to modify the course of HD.

## 2. Physiological Functions of Wild-Type HTT

HTT is a large, multi-domain protein, consisting of 3144 amino acids [19]. It performs multiple essential physiological functions, particularly the critical roles in the development and maintenance of the nervous system. HTT is indispensable for embryonic development, as its knockout in mice results in embryonic lethality [20]. It also regulates gene expression [21], cell adhesion [22], cell survival [23], and intracellular transport [24]. Disruption of HTT’s normal activity contributes to the pathogenesis of HD [25].

Mechanically, HTT enhances the transport of brain-derived neurotrophic factor (BDNF) vesicles [26], autophagosomes in axones [27], TrkB receptors in striatal dendrites [28], amyloid precursor protein (APP) vesicles [29], and synaptic vesicles through GABAR signaling [30]. Beyond its role in intracellular trafficking, HTT also regulates the expression of genes related to neuronal development by modulating histone epigenetic states and altering chromatin structure [31]. In line with this broad regulatory capacity, HTT influences the activity of several transcription factors, specifically, such as REST/NRSF [32]. Moreover, it is involved in synaptic plasticity processes such as long-term potentiation (LTP), influencing learning and memory [33].

## 3. Pathogenic Roles of mHTT

mHTT exerts deleterious effects on wild-type HTT (wtHTT) function through a combination of loss of normal protective roles and gain of toxic activities. Structurally, mHTT retains key interaction domains and competes with wtHTT for binding partners such as HAP1, dynein, and kinesin, disrupting axonal transport of organelles and brain-derived neurotrophic factor (BDNF) vesicles [34], and sequestering wtHTT into insoluble aggregates [35].

Functionally, mHTT exhibits reduced capacity to support neuronal survival [36], regulate transcription via REST/NRSF [32], and facilitate selective autophagy through ULK1 complex modulation [37]. Additionally, mHTT gains novel toxic properties, including aberrant interactions with transcription factors (e.g., CREB, Sp1, p53) leading to transcriptional dysregulation [32], direct impairment of mitochondrial function with calcium leakage and oxidative stress [38] and mitochondrial protein import [39], overload of proteostasis systems such as the ubiquitin–proteasome system (UPR) [40] and autophagy [41], and interfere with the function of lysosome [42]. In summary, mHTT not only diminishes its neuroprotective function of wtHTT, but also activates toxic signaling pathways, and these effects synergistically promote the progression of neurodegeneration in HD.

## 4. Post-Translational Modifications of HTT and Their Functional Implications

Post-translational modifications (PTMs) play a crucial role in shaping the structural dynamics, subcellular localization, interaction networks, and stability of both wtHTT and mHTT proteins, thereby critically influencing their physiological functions and pathogenic potential in HD models. Numerous PTMs have been identified on HTT, including acetylation [13], phosphorylation [14], ubiquitination [43], SUMOylating [16], and palmitoylation [17], each contributing uniquely to its aggregation behavior and toxicity.

HTT protein is organized into multiple functional regions, each contributing to its diverse cellular roles. The N-terminal region of HTT contains the N17 domain, the polyQ tract, and the proline-rich region (PRR). The N17 domain is subject to complicated post-translational modifications, which affect the degradation of mHTT [44], subcellular localization [45], membrane association [46], and aggregation propensity [47]. Loss of N17 leads to enhanced nuclear pathology, transcriptional dysregulation, and more severe disease phenotypes in HD models, highlighting its importance in regulating disease progression [48]. The rest of the HTT protein contains multiple HEAT repeats, which may function as a solenoid-like structure acting as a scaffold for protein complexes and mediating inter- and intra-molecular interactions [18]. Bioinformatic analyses of HTT report HEAT repeats clustered into larger alpha-rod domains separated by disordered regions. It has been revealed that most PTMs were located in clusters within the disordered regions rather than within the predicted α-helical structured HEAT repeats [49].

Mapping and functionally characterizing these modifications not only deepens our mechanistic understanding of disease onset and progression but also identifies promising therapeutic targets for modulating HTT function and mitigating mHTT toxicity. We summarize the current understanding of the major PTMs identified on HTT, their regulatory enzymes, and cross-talk mechanisms.

### 4.1. Phosphorylation Modifications (Summarized in Table 1)

**Figure 1:**
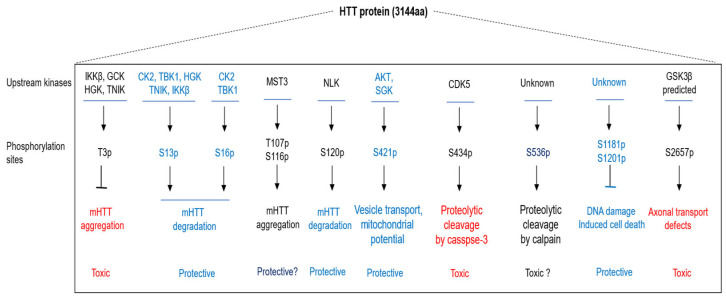
The key phosphorylation sites of the HTT protein. Key phosphorylation sites of the huntingtin (HTT) protein are summarized, including T3, S13, S16, T107, S116, S120, S421, S434, S536, S1181, S1201, and S2657. Functional consequences of these modifications are indicated: red labels denote phosphorylation events associated with neuronal toxicity, whereas blue labels indicate those linked to neuroprotection. Arrows (“↓”) represent enhancing or activating regulatory effects, bars (“
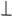
”) denote inhibitory regulation, and question marks (“?”) indicate unclear or yet-to-be-determined roles.

The HTT protein and its phosphorylation are closely linked in both normal neuronal physiology and Huntington’s disease (HD) pathology. Multiple phosphorylation sites of HTT cluster within the N-terminal domain or in predicted proteolytic domains between HEAT repeats. N17 is a hotspot of phosphorylation sites of HTT that have been identified, along with their associated functions. Recent studies have revealed important roles for phosphorylation at additional HTT residues. Here, we provide a systematic summary and analysis of these phosphorylation-dependent functions of HTT and their relations with mHTT cytotoxicity. The key phosphorylations of the HTT protein are shown in Figure 1.

#### 4.1.1. Phosphorylations in the N17 Domain

T3 phosphorylation, identified by mass spectrometry [50] and further validated using a site-specific phospho-antibody, has been implicated in mHTT aggregation and neurotoxicity, while the effects have conflicting reports [46]. Phosphorylation at T3 stabilizes membrane membrane-independent α-helical conformation of the N-terminal 17 amino acids (Nt17) and significantly inhibits the aggregation of mutant Httex1 [46]. Phospho-null mutation T3A and phospho-mimetic mutation T3D exert distinct effects on mHTT aggregation in cellular HD models; both mutations significantly reduce neurodegeneration in a drosophila HD model [50]. In another Drosophila HD model, enhancing T3 phosphorylation by downregulating protein phosphatase 1 (PP1) resulted in elevated neurotoxicity [51]. This paradox indicates that mHTT aggregation does not always correlate directly with toxicity and varies with cellular and molecular context: it is coupled to the modification state of adjacent residues and to the binding/dissociation dynamics associated with lipid membranes. In vitro, semisynthetic mHTT carrying site-specific T3 phosphorylation markedly suppresses the aggregation of mutant Httex1, while acetylation at K6 inhibits the inhibitory effect of T3 phosphorylation [52]. Overall, T3 phosphorylation inhibits the aggregation of mutant HTT, with its functional outcomes being influenced by neighboring modifications.

S13/S16 phosphorylation plays critical roles in regulating HTT aggregation, toxicity [44], and subcellular localization [48]. On one hand, phosphorylation at S13/S16 decreases membrane-induced helicity of the N17 domain, modulating HTT membrane interaction and aggregate formation [53], and thereby reducing aggregate formation and neuronal toxicity [54]. On the other hand, this modification interferes with the N17 nuclear export signal, leading to increased nuclear retention of HTT, which may in turn influence its transcriptional regulatory functions. Single or double phosphorylation at S13/S16 confers neuroprotection in HD models, whereas phosphor-resistant mutations at these sites promote inclusion formation and exacerbate toxicity [51]. IKK phosphorylates Huntingtin and targets it for degradation by the proteasome and lysosome. Phospho-mimetic substitutions at S13 and S16 enhance Hsc70 binding, indicating S13/S16 phosphorylation facilitates CMA-mediated HTT degradation, whereas polyQ expansion weakens this interaction, suggesting mHTT compromises this process and promotes mHTT accumulation [44]. Moreover, upon TBK1 activation, enhanced phosphorylation at S13 has been shown to promote mHTT degradation via the autophagy pathway [55]. However, the efficiency of TBK1-mediated autophagical degradation of mHTT is limited in that TBK1 does not phosphorylate preformed mHTT aggregates and the fibrous or rigid inclusions repel autophagy receptors such as p62, making it difficult for mHTT to effectively engage with autophagosomes and thereby escape degradation [56]. Collectively, phosphorylation at S13 and S16 reduces mHTT aggregation by modulating the N17 conformation and promotes its degradation through proteasome, autophagy, and CMA pathways.

#### 4.1.2. Modulators of HTT Phosphorylation in the N17 Domain

CK2-mediated phosphorylation stabilizes the N17 α-helical structure in a protective conformation, thereby reducing aberrant intermolecular interactions and suppressing the nucleation of toxic aggregates. Inhibition of CK2 results in decreased phosphorylation at S13 and S16, which correlates with enhanced aggregation and cellular toxicity [57]. Conversely, promoting CK2 activity supports a phosphorylation-dependent protective state of HTT [58]. However, despite its therapeutic potential, CK2 is considered a challenging drug target. Since CK2 is ubiquitously expressed and constitutively active, it regulates numerous essential processes such as transcription, apoptosis, and DNA repair [59], global activation of CK2 could therefore result in widespread off-target effects and toxicity. Altogether, the data highlight that only precise interventions, such as selectively targeting CK2–HTT binding or regulating site-specific phosphorylation events, may effectively mitigate the toxic effects of mHTT.

IKKβ has been identified as a critical kinase that phosphorylates HTT at serine residues S13 and S16. Early work demonstrated that IKK-mediated phosphorylation at these sites facilitates the clearance of mutant HTT in the CMA and proteasomal pathway, thereby reducing aggregate formation and cellular toxicity [44]. Subsequent studies further confirmed that IKKβ specifically regulates this modification, with phospho-mimetic mutation at S13/S16 suppressing fibrillization and inclusion body formation, ultimately exerting a neuroprotective effect [57]. It has been reported that IKKβ phosphorylates S13 via IKK complex /NF-κB and IKK-IRF3 pathways, suggesting IKKβ regulates HTT S13 phosphorylation, while chronic NF-κB drive may also reprogram proteostasis toward an inflammatory state and sustained IRF3 signaling may compromise protein homeostasis [60]. Collectively, these findings identify IKKβ-dependent phosphorylation of S13/S16 as a critical modulatory mechanism underlying HTT pathogenicity and a promising therapeutic target for Huntington’s disease. Nonetheless, the broader biological effects associated with sustained IKKβ activation may lead to long-term adverse outcomes.

TBK1 specifically phosphorylates S13 and S16 [46], and also phosphorylates key components of the autophagy pathway, including OPTN, p62/SQSTM1, NDP52, and TAX1BP1 [61]. It has been reported that TBK1 suppresses the early oligomerization and aggregation of mHTT via the autophagy pathway. However, it does not phosphorylate mHTT in fibrils or inclusions, nor does its overexpression clear pre-formed aggregates [56], suggesting TBK1 mainly exerts its effects during the early or pre-aggregation stages.

PP1 acts as the major phosphatase responsible for dephosphorylating T3, S13 and S16. Genetic and pharmacological inhibition of PP1 resulted in lower mHTT aggregation and increased toxicity and T3 major mediator of PP1activity. Furthermore, PP1 functions in direct opposition to CK2, IKKβ, and TBK2, and serves as a critical negative regulator of protective phosphorylation at the N17 domain [51]. Since PP1 is highly expressed in the brain and particularly enriched in medium spiny neurons [62], specific inhibition of PP1 activity may help reduce mHTT aggregation and suppress its cytotoxicity.

#### 4.1.3. Other Phosphorylation Sites on HTT Protein

T107 and S116 phosphorylations were identified by mass spectrometry [63]. It has been demonstrated that STE20-like kinase 3 (MST3) selectively and efficiently phosphorylates T107 and S116 [64]. Phosphor-resistant and mimicking mutations S116A significantly reduced toxicity in primary cortical neurons expressing mHTT [63]. However, it has been argued that phosphorylation-mimicking or resistant mutants cannot reproduce all aspects of bona fide phosphorylation effects. An aggregation kinetics assay in vitro supported that double phosphorylation at T107 and S116 significantly promoted aggregation of mHTT, while single phosphorylation at T107 slowed aggregation of mHTT and phosphorylation at S116 did not affect the aggregation. Overall, phosphorylation at T107 and S116 may exert context-dependent effects that likely interact with proteolytic processing and structural domains beyond Httex1 and their toxicity needs further clarification [64].

S120 phosphorylation in HTT has been identified by mass spectrometry analysis [64]. It has been revealed that Nemo-like kinase (NLK) phosphorylates S120 and reduces soluble mHTT levels, enhances its clearance, and decreases aggregate formation. In neuronal and mouse HD models, this modification alleviated neurotoxicity and was associated with improved motor and cognitive performance. NLK lowers mHTT levels in a kinase activity-dependent manner, while having no significant effect on normal HTT protein levels in mouse striatal cells, human cells and HD mouse models. NLK promotes mHTT ubiquitination and degradation via the proteasome pathway. These results suggest S120 phosphorylation as an allele-selective target to reduce mHTT levels and toxicity [65].

S421 phosphorylation has been identified as a substrate of AKT and was demonstrated by a phospho-specific antibody [66]. Phosphorylation at S421 provides neuroprotection through multiple mechanisms. Phospho-mimetic mutation S421D can restore the transport of brain-derived neurotrophic factor (BDNF)-containing vesicles in mHTT-expressing HD cell models [67], and improve mitochondrial morphology, membrane potential, and oxidative phosphorylation in the neural cell HD model [68]. Akt, activated by IGF-1, is the major kinase that phosphorylates HTT at S421, and mediates neuroprotection in HD models [66]. SGK can phosphorylate HTT at S421 in vitro [69]. It is important that SGK is increased in the striatum of HD patients and plays an important role in maintaining the stability of striatal neurons [69]. In summary, strategies aimed at boosting S421 phosphorylation may provide neuroprotection in Huntington’s disease.

S434 phosphorylation was identified as a CDK5 substrate via in vitro/in vivo kinase assays and confirmed by a phosphorylation site-specific antibody. HTT S434 phosphorylation protects against HTT proteolytic cleavage by caspase-3, aggregation, and downstream toxicity [70]. However, Cdk5-mediated phosphorylation of DARPP-32 at T75 inhibits protein kinase A (PKA) signaling, leading to dendritic spine instability and depressive-like behaviors [71]. Thus, S434 phosphorylation is a critical protective site within the Cdk5–HTT axis, playing a decisive role in cell toxicity. Given the pleiotropic actions of CDK5 on numerous neuronal substrates, the precise regulation of HTT phosphorylation at S434 is crucial to balance its physiological and pathological roles, thereby mitigating the progression of Huntington’s disease.

S536 phosphorylation was identified by mass spectrometry. The S536 residue lies in the proteolytic susceptibility domain of HTT [72]. Calpain-mediated cleavage has been detected in mouse brain, HEK293T cells, and human HD tissues, producing a 536-aa fragment of HTT [73]. S536 phospho-mimic mutation inhibited the production of calpain-derived mHTT fragment and reduced cellular toxicity, while S533 or S535 mutations did not show protective function [14]. Furthermore, calpain-mediated cleavage of mutant huntingtin (mHTT) generates highly toxic N-terminal fragments that readily translocate into the nucleus, where they form aggregates and contribute to cellular toxicity, including transcriptional dysregulation and energy metabolism impairment [74]. Another study demonstrated that the clearance of mHTT exon-1 in cellular models requires calpain activity; inhibition of calpain or lysosomal acidification blocks this process, indicating a dual role of calpain in the regulation of mHTT aggregation [75]. A recent study showed that inclusions cannot be directly engulfed by autophagosomes. Instead, they must first be fragmented by a DNAJB6–HSP70–HSP110–proteasome cooperative system, which generates smaller fragments that are subsequently recognized by p62/SQSTM1 and LC3, thereby facilitating their degradation through aggrephagy [76]. Therefore, it is essential to investigate whether phosphorylation at S536 modulates DNAJB6–HSP70–HSP110–mediated degradation of mHTT aggregates.

S1181/1201 phosphorylation was identified by mass spectrometry and validated by a phosphorylation site-specific antibody [77]. Phosphorylation at these sites shows a marked increase in HD mouse brain compared with wild-type counterparts, suggesting a disease-associated upregulation [78]. It has been demonstrated that CDK5 phosphorylates these two sites, and this modification confers a neuroprotective effect: phospho-deficient mutants (S1181A/S1201A) sensitize cultured striatal neurons to p53-mediated toxicity, whereas phospho-mimetic mutants are protective [77]. However, genetic phospho-ablation at S1181 and S1201 in the mouse showed reduction in anxiety/depression-like phenotypes and increased axonal transport of BDNF, suggesting HTT function is associated with etiology of mood disorders and anxiety/depression in HD [79]. Thus, phosphorylation of HTT S1181/S1210 may activate distinct signaling pathways under different cellular contexts, leading to divergent functional outcomes.

S2114/2116 phosphorylation was identified by mass spectrometry and validated by a phosphorylation site-specific antibody [80]. Mutant HTT (mHTT) exhibits abnormally enhanced binding to PRC2 and disrupts the normal transcriptional profile of neuronal genes, causing transcriptional dysregulation and selective neuronal vulnerability by altering its PRC2 chromatin distribution [31]. S2114 and S2116 residues exhibit increased phosphorylation under the mHTT background, and phospho-deficient mutants (S2114A, S2116A) in the HD model significantly reduce the aberrant enhancement of PRC2 histone methyltransferase activity and alter its chromatin distribution. S2116A additionally restores structural integrity between the N-HEAT and C-HEAT domains. These findings indicate that blocking phosphorylation may attenuate mutant HTT toxicity and chromatin dysregulation [80], while another study, which explored the role of HTT PTM sites on mouse primary neuron toxicity caused by the polyQ expansion using nuclear condensation and mitochondria viability assays, suggests no significant effect of S2116A [63]. Collectively, S2114 and S2116 phosphorylation primarily confer a “gain-of-function” property to mutant HTT, by enhancing PRC2 activity and inducing chromatin compaction and transcriptional repression. It remains unclear whether blocking S2114/S2116 phosphorylation can alleviate mHTT-mediated cytotoxicity.

S2657 phosphorylation was identified by mass spectrometry [78] and is predicted to be targeted by GSK3β and ERK1, but has not been experimentally validated. In vitro kinase phosphorylation assay revealed that both GSK3β and ERK1 regulate phosphorylation of wild type and mHTT. GSK3β-dependent phosphorylation contributes to global axonal transport defects, and tends to promote mHTT aggregation and its mediated toxicity, whereas ERK phosphorylation of HTT suppresses aggregation and exerts neuroprotective effects [81,82]. These contrasting outcomes may be accounted for by the fact that GSK3β typically recognizes substrates through a priming phosphorylation located four residues downstream (at the +4 position) relative to the target site [83]. Given that GSK3β and ERK1 exert opposing effects on mHTT-induced axonal transport defects and neuronal cell death, and that S2657 is a potential shared phosphorylation site of these kinases, this residue may serve as a key regulatory node linking kinase signaling to mHTT pathogenicity. Elucidating the precise biological role of S2657 could provide new insights and identify a potential therapeutic target for Huntington’s disease.

#### 4.1.4. Phosphorylations with Limited Information

Several phosphorylation sites have been identified by independent mass spectrometry analyses, including T271, S417, S431, S432, S457–464, S487/T488, S491, S644, S1181, S1179, S1181, S1201, S1350, S1864-S1876, S2076, S2337, S2342, S2489, S2550, S2615, S2653, and S2639 [14,78,84,85], while lacking validation by site-specific antibodies or in vitro/in vivo kinase assays. Some of them cluster within proteolytic domains of the HTT protein and were screened for their functions in modulating cell toxicity and mitochondrial abnormalities caused by the mHTT protein in primary neurons [63], and some did not exhibit significant functional effects in the neuronal toxicity assays [84].

While the studies provide useful insights, the conclusions about phosphorylation-dependent regulation of mHTT toxicity are weakened by the exclusive reliance on phospho-mimetic and phospho-resistant mutations without direct biochemical validation [86]. This limitation raises concerns about whether the observed effects truly reflect bona fide phosphorylation or instead result from structural artifacts of the mutations. Further, some assays used exogenous mHTT protein expressed in 293T cell lines [84,85] and one specific essay used systematic identification and quantification of post-translational modifications in endogenous full-length HTT protein derived from HD knock-in Q175 mice and human HD post-mortem brain tissue [78]. Notably, Ratovitski’s study revealed a considerable number of phosphorylation sites located in the C-terminal region of HTT. This finding suggests that, given HTT is a protein with 3144 amino acids, phosphorylation modifications in the C-terminal region may play important roles in regulating its normal physiological functions and homeostasis [78]. Further exploration of how these sites are regulated, both in terms of their impact on normal physiology and their contribution to mHTT pathogenicity, will help identify new therapeutic targets for Huntington’s disease.

**Table 1:** List of selected phosphorylation sites with established functional information.

Residue	Function	Kinase	Phosphatase	Identification/Validation Method	References
T3	Inhibits mHTT aggregation and decreases mHTT toxicity	IKKβ, GCK, HGK, TNIK	Not reported	MS/Ab/Phos-Tag SDS-PAGE	[46,87]
S13	Inhibits mHTT aggregation, enhances mHTT degradation, and decreases mHTT toxicity	CK2, TBK1, HGK, TNIK, IKKβ	Not reported	MS/Ab	[46,60]
S16	Inhibits mHTT aggregation, enhances mHTT degradation, and decreases mHTT toxicity	CK2, TBK1	Not reported	MS/Ab	[46]
T107/S116	Double phosphorylation enhances mHTT aggregation	MST3	Not reported	ESI-MS/UPLC/Ab	[64]
S120	Increases mHTT degradation and decreases aggregation and toxicity	NLK	Not reported	MS/in vitro kinase assays	[64,65]
S421	Maintains mitochondrial potential and anterograde/retrograde vesicle transport.	AKT, SGK	PP1 and PP2A	MS/Ab	[67,68,78,82,88,89]
S434	Decreases caspase-mediated mHTT cleavage and mHTT toxicity	CDK5	Not reported	MS/Ab	[70,77,89]
S536	Decreases calpain-mediated mHTT cleavage and cytotoxicity	Not reported	Not reported	MS	[14]
S1181/S1201	Protects the cell from DNA damage-induced cell death, and dephosphorylation reduces mood disorders	CDK5	Not reported	MS/Ab	[14,77,78,82]
S2114	Enhances mHTT and PRC2 interaction	Not reported	Not reported	MS/Ab	[14,80]
S2116	Enhances mHTT and PRC2 interaction, disrupts normal conformational stability.	Not reported	Not reported	MS/Ab	[14,80]
S2657	Regulates axonal transport and mHTT-mediated neuronal cell death	ERK, GSK3β	Not reported	MS/in vitro kinase assay	[82]

### 4.2. Acetylation

Acetylation of lysine residues is a reversible modification primarily catalyzed by histone acetyltransferases (HATs), such as CBP and P300, and removed by histone deacetylases (HDACs) or sirtuins (SIRTs). It has been identified that the HTT protein contains multiple acetylation sites, including K6, K9, K15, K178, K236, K345, K444, K815, k818, K1190, k1204, k1246, K1875, k2548, k2615, and k2969 [53,78,90]. For several lysine residues, the underlying modification mechanisms and their associated biological functions have been investigated in considerable detail. These studies provide valuable insights into how acetylation modifications influence HTT’s structural dynamics, cellular interactions, and contribution to neuronal survival or toxicity. The following paragraphs provide a detailed summary and discussion of these findings. However, the modification of K178, K236, K345, K815, k818, K1190, k1204, k1246, k1875, k2548, k2615, and k2969 residues requires further validation.

K6/9/15 acetylation Chemically induced K6, K9, and K15 acetylation reduces the toxicity of mHTT by promoting the formation of globular mHTT aggregation and the membrane binding affinity [91]. The functions of acetylation at K6 and K9 are distinct. K6 acetylation is involved in enhancing the aggregation of HTTex1by reversing the effect of T3 phosphorylation [52], whereas K9 acetylation and the associated S13/S16 phosphorylation contribute to promoting mHTT clearance through the autophagy–lysosomal degradation pathway [44]. These results indicate that the interplay between acetylation and other post-translational modifications plays a crucial role in the regulation of HTT structure and degradation.

K444 acetylation has been reported to promote autophagic clearance of mHTT and reverses the toxic effects of mHTT in primary striatal and cortical neurons and in a transgenic C. elegans model of HD. CREB-binding protein (CBP) has been demonstrated to be the main acetylase that enhances K444 acetylation, and HDAC1 is the main deacetylase of K444ac [13]. Inhibitors of HDAC1 (such as SAHA [92] and sodium butyrate [93]) show protective effects in HD models.

One study reported that the acetylation-resistant mutant K444R enhanced cell death in cultured cortical and striatal neurons [13], whereas another found that the same mutation reduced neuronal toxicity [63]. These divergent outcomes are likely due to differences in experimental systems: the first employed full-length mHTT containing 82 glutamines (82Q), while the second used an N-terminal fragment, indicating regulatory elements present in the full-length HTT protein may influence how the K444R mutation modulates mHTT-induced toxicity. Combined use of mass spectrometry and antibody-based detection has confirmed that acetylation at K236, K345, and K444 is more prominent in mHTT compared with WT, while a multiple reaction monitoring (MRM)-MS quantitation assay revealed that acetylation at K178 is significantly higher in wtHTT. The differential acetylation patterns between wtHTT and mHTT suggest that abnormal acetylation contributes to mHTT pathogenicity by influencing its aggregation, degradation, and interaction networks [90]. Targeting these residues with site-selective modulators of acetylation could provide a means to fine-tune mHTT activity, thereby offering novel therapeutic strategies for Huntington’s disease.

### 4.3. Ubiquitination

Ubiquitination regulates protein homeostasis by tagging substrates with ubiquitin chains of distinct linkages. K48-linked chains generally direct proteins to proteasomal degradation, while K63-linked chains mediate non-proteolytic functions such as signaling and autophagy. By coordinating the ubiquitin–proteasome system and autophagy, ubiquitination plays a crucial role in maintaining proteostasis, and its dysregulation is strongly implicated in neurodegenerative diseases [94].

In the cerebral cortex of HD patients, mHTT inclusion bodies (IBs) are detected in both the cytoplasm and the nucleus and exhibit marked structural and immunophenotypic heterogeneity. Notably, the HTT species within these inclusions are extensively ubiquitinated, with K63-linked polyubiquitin chains occurring more frequently than the classical K48-linked type [95]. The ubiquitination state of HTT not only reflects its aggregation propensity but also modulates the toxicity of inclusions. Elucidating these mechanisms is crucial for understanding the pathogenesis of HD and for identifying novel therapeutic strategies. Five ubiquitination sites have been identified in the HTT protein, including K6, K9, K132, K804, and K837 [15].

#### 4.3.1. Ubiquitinations That Have Specific Functions

K6 and K9 were identified as specific ubiquitination sites of mutant HTT in striatal and cortical tissues in HD mouse and rat models by mass spectrometry. Elimination of the N-terminal ubiquitination sites (K6 and K9) in mHTT exon 1 constructs markedly delayed the onset of aggregate formation, reduced mean aggregate size, and concomitantly increased the number of oligomeric mHTT, which induced cell death in primary cortical neurons and cultured cells [96]. Mutation of mHtt K6 and K9 is associated with the appearance of numerous nuclear aggregates and suppresses the molecular dynamics of aggregate-associated mHtt [97]. In contrast, K6R or K9R mutations in full-length HTT 82Q did not exhibit increased cell death in primary cortical and striatal neurons, suggesting that the functional consequences of these ubiquitination events differ between the exon 1 fragment and the full-length protein [63]. K132, K804, and K837 were identified as specific ubiquitination sites of wtHTT by mass spectrometry, among which K132 and K804 represent the predominant sites [15]. Future studies should aim to identify the specific E3 ubiquitin ligases and deubiquitinases (DUBs) that selectively act on K6/K9, in order to determine whether these sites represent actionable targets for therapeutic development. Elucidating the mechanisms and critical functions of modifications at these two sites will provide important insights into the regulation of HTT homeostasis and the pathogenesis of HD, and may offer new targets for therapeutic drug development.

#### 4.3.2. HTT Related E3 Ligase

E3 ubiquitin ligases are central regulators of the ubiquitin–proteasome system (UPS). They are responsible for transferring activated ubiquitin onto specific substrate proteins. By providing substrate specificity and determining ubiquitin chain topology, E3 ligases dictate the cellular fate of ubiquitinated proteins. In Huntington’s disease (HD), E3 ligases play a critical role in regulating the turnover of mHTT and determining whether the protein is efficiently cleared or accumulates into toxic aggregates. Several E3 ligases have been implicated in HTT metabolism.

CHIP is a C-terminal Hsp70-interacting protein and functions as both a co-chaperone and an ubiquitin ligase, thereby bridging molecular chaperones and the ubiquitin–proteasome pathway. CHIP targets multiple polyglutamine (polyQ) proteins, including HTT, ataxin [98] and androgen receptor (AR) [99] and ataxin, in a chaperone-dependent manner. It specifically promotes K48-linked ubiquitination of misfolded HTT, thereby enhancing proteasomal degradation and reducing aggregate burden. Importantly, CHIP haploinsufficiency was reported to exacerbate the disease phenotype in an HD mouse model, providing further evidence for its protective role in HD pathogenesis [100].

HRD1 is an ER-associated E3 ubiquitin ligase that promotes the ubiquitination and proteasomal degradation of misfolded or aggregation-prone proteins and inhibits Caspase-3-mediated apoptosis [101]. HRD1 can interact with the N-terminal fragment of HTT in a polyQ length-independent manner and preferentially directs mHTT for degradation. HRD1 interacts with the N-terminal 588–amino acid fragment of HTT in a manner with depends on its catalytic activity, suggesting that HTT may undergo ubiquitination within its N-terminal domain. Thus, HRD1 reduces aggregate formation, alleviates proteotoxic stress, and protects neuronal cells, underscoring its biological significance as a potential therapeutic target in Huntington’s disease [102].

Parkin, an E3 ubiquitin ligase, facilitates the ubiquitination and degradation of expanded polyglutamine proteins. Its overexpression suppresses polyQ protein aggregation and cytotoxicity, alleviates proteasome dysfunction, and reduces caspase-12 activation [103]. However, the role of parkin in HD models appears more complex. In a Drosophila model of HD, enhanced PARKIN expression restored mitochondrial function and significantly ameliorated neurodegeneration, improving both viability and lifespan. In R6/1 mice, partial suppression of parkin decreases the number of visible mHTT inclusions but is associated with increased apoptosis and elevated autophagy markers [104]. The data suggest the toxic species of mHTT responsible for cell death remains to be determined and the precise role of parkin in HD pathogenesis requires further investigation.

SCF (Skp1–Cullin–F-box) E3 ubiquitin ligase complex plays an essential role in polyQ diseases by targeting the polyglutamine-expanded protein for ubiquitination and subsequent proteasomal degradation. Dysregulation of SCF complex components, particularly Skp1, Cul1, and specific F-box proteins, leads to the accumulation of toxic HTT aggregates, disruption of protein homeostasis, and enhanced neuronal dysfunction in HD mouse and drosophila models [105]. A noteworthy finding is that the core components of the SCF complex are reduced across multiple Huntington’s disease models [106]. These results imply that reduced levels of the SCF complex might contribute to polyQ disease pathology. Although SCF family ligases may influence mutant HTT turnover, direct evidence of SCF complex targeting polyQ-expanded proteins remains to be demonstrated.

UBE3A mediates K48-linked polyubiquitination of HTT, thereby targeting it for proteasomal degradation and controlling intracellular protein levels [95]. Evidence from cellular and animal models indicates that reduced UBE3A expression or activity impairs mHTT clearance [107], leading to enhanced aggregate formation, accelerated disease phenotypes, and shortened lifespan, whereas maintaining UBE3A function can mitigate proteostasis defects associated with HD [108]. Although the specific ubiquitination sites on HTT remain unidentified, UBE3A may target lysine residues K6 or K9 of mHTT, as it interacts with the N67–150Q fragment of the protein [109]. Studies have demonstrated that UBE3A is strongly recruited into nuclear mHTT aggregates in HD mouse models, in contrast to its predominantly soluble distribution in wild-type mice, suggesting a potential loss of its physiological function [110]. Collectively, the data support a protective role of UBE3A in HTT metabolism, yet a more thorough elucidation of its functions and mechanisms is required to fully assess its value as a therapeutic target.

### 4.4. SUMOylation

SUMOylation involves the addition of small ubiquitin-like modifiers (SUMO) to lysine residues, often regulating nuclear–cytoplasmic trafficking, stability, and transcriptional activity. In the context of Huntington’s disease (HD), SUMOylation occurs predominantly at K6 and K9 of mHTT, which overlap with ubiquitination sites (e.g., K6 and K9 in HTT exon 1). Studies have shown that SUMO-1 can increase mHTT solubility and toxicity [111], whereas SUMO-2 has been found to accumulate in the striatum and is associated with the pathogenic aggregation of mHTT [45], suggesting that the role of SUMOylation in mHTT aggregate formation varies depending on the specific SUMO modification and SUMOylation may not simply regulate aggregation but could shift mHTT toward distinct conformational states with different toxic properties. Multiple research have revealed that in cell, Drosophila, and mouse HD model, genetic reduction in SUMO activity or exogenous expression of sumo or ub resistant mutants, mHTT K6,9R, significantly reduced cytotoxicity [112]. Multiple biochemical and mutagenesis studies have identified K6, K9, and K15 as a clustered region serving as the predominant SUMO acceptor sites, mediated by the E3-like activities of Rhes [111] and PIAS1 [45]. Selective targeting of SUMO pathways could help reduce the harmful forms of mHTT without completely abolishing HTT functions, which is essential for cellular homeostasis.

### 4.5. Palmitoylation Modification

Palmitoylation of HTT is crucial for its normal distribution and function. Specific palmitoylation at the C214 residue of HTT was originally demonstrated by radioactive metabolic labeling [17]. This modification is catalyzed by the palmitoyl acyltransferases huntingtin-interacting protein 14 (HIP14) and HIP14-like (HIP14L), both of which have been implicated in the regulation of HTT trafficking and stability [113]. In HD models, mHTT suppresses the interaction between mutant HTT and HIP14, resulting in reduced palmitoylation levels. Removal of palmitate catalyzed by the acyl-protein thioesterase 1 (APT1) decreases C214 palmitoylation. Multiple studies showed that inhibition of APT1 activity reduces mHTT aggregation and mHTT-induced cytotoxicity in the primary neuronal HD cell model [113] and an HD mouse model [114]. These findings highlight dysregulated palmitoylation as a critical contributor to mHTT toxicity, and precise enhancement of C214 palmitoylation may represent a promising therapeutic strategy for HD.

### 4.6. Crosstalk Among PTMs

Different post-translational modifications (PTMs) targeting the same amino acid residues or occurring at adjacent sequences within the HTT protein, can exert distinct or even opposing effects on HTT aggregation and toxicity, depending on the specific cellular context. Understanding PTM crosstalk in shaping the molecular pathogenesis of Huntington’s disease may provide new avenues for therapeutic intervention.

**Figure 2:**
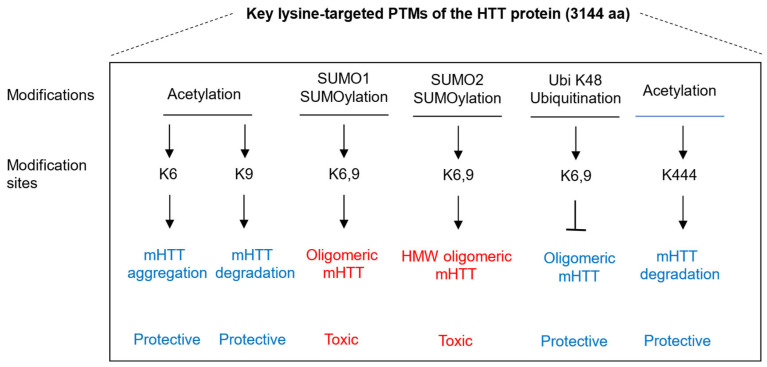
The key lysine-targeted post-translational modifications (PTMS) of HTT protein. Key lysine residues of the huntingtin (HTT) protein are summarized, including K6, K9, and K444, with the corresponding post-translational modifications indicated above each residue. Functional consequences of these modifications are indicated: red labels denote phosphorylation events associated with neuronal toxicity, whereas blue labels indicate those linked to neuroprotection. Arrows (“↓”) represent enhancing or activating regulatory effects, and bars (“
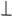
”) denote inhibitory regulation.

Competition of PTMs, such as acetylation, ubiquitination, and SUMOylation at the same lysine, constitutes an important regulatory mechanism that modulates HTT stability and aggregation. The key lysine-targeted PTMS are shown in Figure 2. Research on the interplay between SUMOylation and ubiquitination in HTT has focused on the Nt17, particularly the lysine residues K6 and K9, and K15. Evidence shows that SUMO and ubiquitin can compete for the same lysine residues, and such competitive modification exerts opposing effects on mutant HTTex1 toxicity. In Drosophila HD models, SUMOylation at these Nt17 lysines exacerbates neurodegeneration, whereas ubiquitination promotes clearance and reduces toxicity [16]. These findings highlight K6 and 9 in Nt17 as pivotal regulatory nodes where SUMOylation and ubiquitination crosstalk modulates HTT stability, aggregation, and toxicity. Acetylation neutralizes the positive charge of lysine, thereby preventing ubiquitin and SUMOylation conjugation at the same site [115]. Acetylation at K444 directs mHTT to autophagic clearance in a p62-dependent way [13], and p62 functions to recruit autophagosomal components to the polyubiquitinylated protein aggregates [94]. Taken together, these findings strongly suggest that acetylation, ubiquitination, and SUMOylation compete for the same lysine residues, thereby determining the degradation route of mHTT and regulating cellular homeostasis.

Proximal PTMs can either enhance or antagonize the effect of individual modifications, thereby fine-tuning protein function. Studies have shown that phosphorylation within the Nt17 region significantly influences other modifications of HTTex1. Phosphorylation at T3 is a critical regulatory factor within Nt17 that suppresses mHTTex1 aggregation, whereas acetylation at K6 antagonizes this phosphorylation-mediated HTT aggregation [52]. Acetylation of HTT at K9 and phosphorylation at S13, leading to elevated nuclear translocation of mHTT protein [44]. Phospho-mimetic mutations (S13,16D) alter the kinetics of SUMO-1 conjugation, leading to faster SUMOylation [45]. Steffan et al. (2004) reported that lysines in the Nt17 domain can be modified by both SUMO and ubiquitin [16], while phosphorylation alters the local conformation [53], shifting the balance between ubiquitination and SUMOylation. In summary, phosphorylation may alter the helical conformation of Nt17, thereby modulating the accessibility of neighboring lysines to various modifications. Thus, it will be essential to consider strategies that simultaneously modulate multiple PTMs to preserve the physiological functions of wild-type HTT while suppressing the expression and cytotoxicity of mHTT. Such multi-site regulatory approaches may provide a more balanced and effective means of mitigating HD progression.

## 5. Pharmacological Modulation of PTMs

The modulation of HTT PTMs has emerged as a promising avenue for therapeutic intervention in Huntington’s disease (HD) [18]. Unlike genetic therapies that aim to silence wtHTT and mHTT expression, PTM-based strategies seek to reshape the conformation, localization, solubility, and degradation fate of mHTT without abolishing total HTT function. This is an especially important consideration given HTT’s essential roles in development and adult neuron maintenance [116]. This section evaluates current pharmacological efforts and key challenges in targeting PTMs to mitigate HD pathogenesis.

### 5.1. HDAC Inhibitors

Sodium butyrate [93], SAHA (vorinostat) [92], and phenylbutyrate [117] have demonstrated neuroprotective effects in R6/2 and N171-82Q mouse models. Improvements observed include delayed symptom onset, increased BDNF levels, and reduced aggregate burden. Some HDAC inhibitors have entered clinical trials, though non-specificity and toxicity remain concerns. SAHA downregulated HDAC2 and HDAC4 protein levels and repressed HDAC7 and HDAC11 mRNA levels in WT and HD mouse models, suggesting it is lack of target specificity [118]. Several HDAC inhibitors have been hindered in their translation into clinical therapies due to their non-specific actions and unpredictable toxicity [119,120]. Thus, only inhibitors that can specifically modulate HTT acetylation may effectively slow the progression of Huntington’s disease and protect neuronal cells.

### 5.2. Kinase Modulators

CEP-1347 is a mixed lineage kinase (MLK) inhibitor that involves blocking the MLK → JNK signaling pathway to suppress mutant huntingtin-induced pro-apoptotic responses, and maintaining or enhancing the MLK → MEK → ERK prosurvival pathway. As a result, it reduces neuronal death and restores brain-derived neurotrophic factor (BDNF) levels. In HD cell models and R6/2 mice, CEP-1347 has been shown to attenuate toxicity and improve HD phenotypes [121]. However, the large-scale clinical testing of CEP-1347 in patients with Parkinson’s disease (the PRECEPT trial) revealed it was safe but had no significant neuroprotective effect [122]. There have been no large-scale clinical trials in HD patients; therefore, direct evidence regarding efficacy is still required.

PD169316-mediated inhibition of p38 PMAPK pathway increases proteasomal chymotrypsin-like activity, reduces accumulation and soluble forms of mHTT, and alleviates HAP40-deletion induced cytotoxicity. It is well established that p38 MAPK is excessively activated in the striatum of HD model, where it contributes to impairment of the ubiquitin–proteasome system, and synaptic dysfunction [123]. However, it is still unclear which direct substrates of p38 are specifically regulated in the HD context, as most current evidence is indirect. Furthermore, whether p38 directly modulates mHTT aggregation through transcriptional regulation or post-translational modification is not fully understood [124]. Finally, the distinct contributions of the individual p38 isoforms (α, β, γ, δ) to disease mechanisms have yet to be delineated, leaving open the possibility that isoform-specific targeting could offer therapeutic advantages.

SP600125 is an ATP-competitive small-molecule inhibitor of c-Jun N-terminal kinase (JNK), and has been shown to reduce mHTT-induced toxicity in several models [121]. JNK is a member of the MAPK family and plays an important role in stress responses, inflammation, and apoptosis. It has been shown that the expression of mutant huntingtin (mHTT) can activate JNK, thereby promoting c-Jun-mediated transcriptional activation and pro-apoptotic pathways [125]. Therefore, inhibition of JNK is considered a potential strategy to alleviate mHTT-induced cytotoxicity [124]. While SP600125 inhibits the kinase activity of JNK, it is not entirely selective. Moreover, most supportive data come from cell and Drosophila models, and systematic efficacy studies in mammalian models such as R6/2 or knock-in mice are scarce. JNK not only mediates pro-apoptotic pathways but may also exert protective effects under certain stress conditions, raising concerns that long-term inhibition could be detrimental [126]. Targeting the JNK pathway in Huntington’s disease requires a more refined approach than global kinase inhibition. In particular, it will be crucial to identify and characterize inhibitors that act on specific downstream substrates of JNK. At the same time, clarifying the molecular mechanisms through which mHTT triggers JNK activation remains an important open question. Moreover, a deeper understanding of the distinct contributions of individual JNK isoforms and their substrate preferences may help guide the design of more selective therapeutic strategies with reduced off-target effects.

IGF-1, through the IGF-1R/PI3K/Akt signaling pathway, directly promotes the phosphorylation of HTT at the S421 site, thereby restoring its regulatory function in vesicle transport and ameliorating neuronal toxicity in the context of mHTT [67]. However, its drawbacks include challenges in crossing the blood–brain barrier [127], lack of specificity, and potential systemic side effects [128].

### 5.3. Palmitoylation Enhancer

ML348 restores HTT-dependent BDNF transport and synaptic function by inhibiting APT1 and enhancing protein palmitoylation, exhibiting robust neuroprotective effects, thereby representing a promising candidate for HD therapy. However, because its primary mechanism is to improve intracellular trafficking and synaptic signaling rather than directly lowering mutant HTT expression, it may need to be combined with complementary HTT-lowering approaches [114].

Palmostatin B (PalmB) is a cell-permeable chiral acyl-β-lactone that primarily inhibits APT1 and APT2, thereby reducing the depalmitoylation of HTT. This inhibition increases the palmitoylation level of mutant HTT (mHTT), and treatment with PalmB has been shown to restore mHTT palmitoylation while reducing aggregation and cytotoxicity in COS-7 cells, YAC128 striatal–cortical neurons, and patient-derived lymphoblasts [113]. However, PalmB shows low selectivity, and it partially inhibits fatty acid synthase (FASN), PNPLA6, and several ABHD proteins, raising concerns about potential off-target effects [129]. Moreover, since protein palmitoylation broadly regulates synaptic proteins such as SNAP25 and PSD95 [130], global enhancement of palmitoylation may perturb synaptic function and network stability [131].

## 6. Can Modulating Post-Translational Modifications Functionally Reverse mHTT Toxicity?

Recent work shows that the MutSβ complex (MSH2–MSH3) is a principal driver of somatic HTT CAG-repeat expansion, with vulnerable human striatal neurons exhibiting higher MSH2/MSH3 levels and HD models [132]. Moreover, lowering MSH3—via genetic ablation, dose-dependent knockdown, or ASO treatment—significantly suppresses somatic expansion (often without obvious safety liabilities in models), underscoring MSH3 inhibition as a promising therapeutic strategy for HD.

Ibañez et al. reported that the population frequency of pathogenic repeat expansions, including the HTT CAG expansion, is significantly higher than expected from clinical prevalence. This discrepancy arises because many carriers remain undiagnosed or asymptomatic due to reduced penetrance, late onset, or limited genetic screening in the general population, suggesting that substantial polyQ expansion often accumulates long before the onset of motor and psychiatric symptoms [133]. Therefore, inhibiting mHTT expression and enhancing its degradation constitute key therapeutic approaches for mitigating the progression of Huntington’s disease (HD).

In HD patients, most neurons have an innocuous but unstable HTT gene, with CAG repeats expanding somatically from 40 to 45 to 100–500+ CAGs in striatal projection neurons (SPNs). SPNs with 150–500+ CAGs lost the ability to repress the expression of senescence/apoptosis genes and leading to cell death [134]. This type of somatic expansion can be detected through blood testing [135]. In a cohort of 10,921 individuals with Huntington’s disease, 28 (≈0.3%) were identified as homozygous for the expanded HTT allele. These individuals exhibited similar ages at onset and disease progression to heterozygous patients, suggesting that a single mutant HTT allele is sufficient to cause the disease [136]. Non-selective reduction in HTT lowers mHTT levels but simultaneously suppresses the normal expression of wild-type HTT, which impairs the maturation and synchronized synaptic activity of human cortical neuronal networks [137]. Thus, a major therapeutic goal is to eliminate mutant HTT while preserving the normal functions of wild-type HTT. Post-translational modifications (PTMs) provide molecular switches that may enable this selective regulation.

Recent preclinical investigations into HD therapeutics have provided encouraging results from two strategies: gene silencing and splicing modulation. Both AMT-130 [138] and PTC518 [139] reduce HTT gene expression at the RNA level in a non-allele-specific manner, thereby lowering the accumulation of mHTT and attenuating its neurotoxicity, but also decreases the level of normal HTT. These data support that therapeutic strategies aiming to decrease the toxic burden of mHTT are effective in slowing disease progression [140,141]. However, the physiological functions of HTT are indispensable, and in the adult nervous system, wild-type HTT contributes to critical processes such as axonal transport of BDNF-containing vesicles [67], synaptic plasticity [142], transcriptional regulation [36], and autophagic clearance [143]. Consequently, long-term and substantial reduction in HTT mRNA may disrupt these normal functions and lead to non-specific toxicity [116,144]. Beyond the central nervous system, HTT is also expressed in peripheral tissues, including the liver, heart, pancreas, and immune cells. Persistent lowering of HTT in these organs could cause metabolic disturbances, immune dysfunction, and other systemic side effects [145]. Recently, several Proteolysis Targeting Chimera (PROTAC) molecules have been identified to selectively lower the toxic oligomeric species of mHTT without enhancing aggregate formation and accumulating cleavage fragments, thereby representing a favorable approach for mitigating pathogenic protein species. However, the long-term consequences for autophagy homeostasis remain unclear [146].

These findings indicate that, despite their respective unresolved limitations, reducing HTT expression or enhancing the selective clearance of mHTT represents an effective strategy to alter the course of HD, providing a solid experimental basis for future clinical translation and therapeutic interventions.

Multiple lines of experimental evidence support the notion that altering post-translational modifications (PTMs) of HTT/mHTT can significantly affect disease progression in HD models. Phosphorylation provides several examples: phosphorylation at S421 maintains the normal function of the endosome and lysosome and improves neuronal survival [24], while phosphorylation at S13/S16 or S116 modulates aggregation and toxicity in HTT N-terminal fragment models [57]. Acetylation at K444 has been shown to promote autophagic clearance of mutant HTT, thereby reducing aggregate burden [13]. Ubiquitination at K6/K9 favors the formation of larger, less toxic inclusions, suggesting a protective sequestration mechanism [15]. Conversely, certain PTMs, such as SUMOylation at the N-terminus, can exacerbate aggregation and cytotoxicity [16]. Importantly, pharmacological interventions targeting enzymes that regulate these modifications, such as kinase activators [55,67], HDAC inhibitors [118,147], or Ubi E3 ligase modulator [148]—have demonstrated beneficial effects in cellular systems and transgenic mouse models.

Collectively, these findings indicate that manipulating PTMs synergistically can delay disease onset, mitigate neurodegeneration, and improve behavioral outcomes in HD models. However, the effects are site- and context-dependent, highlighting the need for systematic studies on full-length HTT to define modification patterns that are consistently protective across disease stages.

## 7. Research Limitations and Future Perspectives

While the field has made significant advances in understanding how post-translational modifications (PTMs) affect Huntingtin (HTT) aggregation and toxicity, a number of critical gaps remain.

### 7.1. Limitations of Experimental Models in Huntington’s Disease Research

Experimental models of Huntington’s disease (HD) are highly diverse and include HTT fragment transgenic models (e.g., R6/2), full-length transgenic models (e.g., YAC128, BACHD), knock-in models (e.g., Q175, zQ175), as well as iPSC-differentiated neuronal models. These models differ systematically in terms of endogenous versus exogenous HTT expression, the length of the genetic construct, affected brain regions and cell types, age of onset and rate of disease progression. Cellular and mouse models expressing mHTT exon-1 fragments have been widely used to study HD. Recent structural studies revealed that the atomic structure of mHTT exon-1 fibrils is highly compact, which restricts the accessibility of post-translational modification (PTM) enzymes or antibodies larger than ~0.8 nm to key N-terminal residues. Consequently, such fragment-based models may mask important physiological roles of PTMs [149]. The heterogeneity of different models substantially influences the interpretation of the role of specific PTMs in HD pathogenesis and has contributed to the lack of consistency in reported outcomes for individual PTM sites or modifications [18,150,151]. Thus, all findings need to be cross-validated across different model systems. It is important to prioritize the use of full-length knock-in (KI) models and iPSC-derived systems and combine site-specific antibodies with PTM-enrichment proteomics (DIA/PRM) to confirm the role of specific PTMs.

Multiple post-translational modifications (PTMs) act on wild-type HTT, including phosphorylation at S421 [67], phosphorylation at S13/S16 [55], and palmitoylation at C214 [17]. These modifications can regulate HTT aggregation, trafficking/clearance, and neurotoxicity, highlighting the PTM-dependent control of HTT’s conformational and functional states. At present, there are no reports of mouse models in which dysregulated PTMs of wild-type HTT lead to HD-like pathology. Recent studies have shown that in striatal projection neurons (SPNs) of HD patients, the CAG repeat sequence of the human HTT gene can undergo somatic expansion from 40 to 45 to more than 100–500 repeats, indicating that the human HTT gene is highly unstable [134]. In contrast, the endogenous mouse Htt gene usually contains only about seven CAG repeats, with an extremely low probability of expansion, thus exhibiting high genetic stability. Therefore, by screening knockout or knock-in mouse models targeting specific PTM-regulatory genes, it may be possible to establish non-polyQ-dependent HD-like animal models, which will help further explore the role of post-translational modifications in the onset and progression of the disease.

### 7.2. Limitation of PTM-Mimetic or PTM-Null Mutants

Accumulated studies have used PTM-mimetic or PTM-null mutants to investigate the function of PTMs in HTT. Although mutation mimics can rapidly provide mechanistic clues, their irreversible nature, lack of specificity, and model dependence may mislead the interpretation of PTM physiological functions. Chiki et al. used protein semisynthesis to investigate the effects of different PTMs. The result shows phosphorylation at T3 stabilizes the α-helical conformation of N17 and significantly inhibits the aggregation of mutant Httex1, while phosphor-mimetic T3D mutation had no effect on N17 conformation and resulted in only intermediate inhibition of Httex1, demonstrating that this mutant does not reproduce the structural and aggregation properties of authentic phosphorylation [152]. In parallel, another study reveals that the phospho-null mutation T3A and the phospho-mimetic mutation T3D exert distinct effects on mHTT aggregation in cellular HD models, while both mutations significantly reduce neurodegeneration [50]. Thus, the conclusions drawn from these approaches should be interpreted with caution and corroborated by complementary methods such as site-specific chemical modification, mass spectrometry, and the generation of knock-in animal models.

### 7.3. Limited Understanding of the Interaction Between Striatum Metabolism and PTM

The striatum and cerebral cortex represent the principal site of early and severe pathology in HD [1] and projection neurons in the striatum and the cerebral cortex are major regions of the HD pathology [153]. Multiple mechanisms contribute to this selective striatal vulnerability, including enhanced susceptibility to excitotoxicity mediated by NMDA receptors [154], impaired mitochondrial Ca^2+^ handling and bioenergetic stress [155], and deficits in cellular stress response pathways [153].

Systematic knowledge of PTM sites, their functions, underlying mechanisms, and disease relevance under the metabolic environment of the striatum will enable the development of targeted interventions, provide biomarkers for treatment monitoring, and guide the design of more selective, context-specific therapies that preserve essential HTT functions while mitigating mutant toxicity.

## 8. Summary and Future Outlook

**Figure 3:**
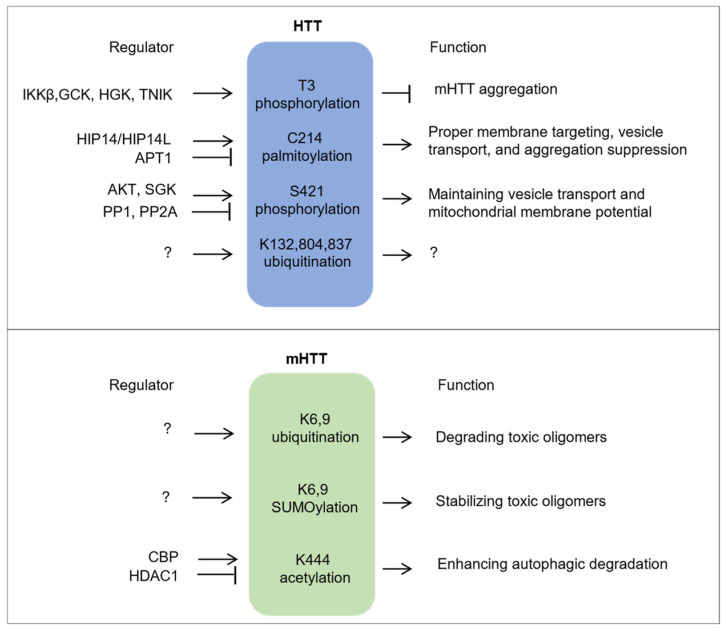
Differential Post-Translational Regulation of Wild-Type and Mutant Huntingtin as a Basis for Allele-Specific Therapy. The schematic diagram illustrates PTMs that are specific to or significantly different between HTT and mHTT. PTMs specific to or predominantly enriched in HTT are shown in a blue rounded rectangle, while those in mHTT are shown in green. “→” indicates enhancing regulatory effects, “
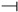
” represents inhibitory regulation, and “?” denotes unclear or yet-to-be-determined regulatory mechanisms or functions.

HTT and mHTT exhibit overlapping yet distinct post-translational modification (PTM) profiles. The schematic diagram illustrates PTMs that are specific to or significantly different between HTT and mHTT, shown in Figure 3.

Despite extensive progress, the HTT PTMs remain in a nascent phase with substantial potential for expansion. To date, efforts have largely centered on isolated modifications in simplified models, leaving a fragmented picture of how PTMs interact and function in the complex environment of the human brain. A comprehensive PTM atlas of HTT—capturing modification dynamics, interaction networks, and functional outcomes in disease-relevant contexts—will be critical for realizing the full potential of PTM-targeted interventions in Huntington’s disease. Integration of advanced proteomics, structural biology, and single-cell techniques promises to resolve longstanding uncertainties and identify novel therapeutic targets.

## Data Availability

No new data were created or analyzed in this study. Data sharing is not applicable to this article.
